# Using Multiscale Entropy to Assess the Efficacy of Local Cooling on Reactive Hyperemia in People with a Spinal Cord Injury

**DOI:** 10.3390/e21010090

**Published:** 2019-01-18

**Authors:** Fuyuan Liao, Tim D. Yang, Fu-Lien Wu, Chunmei Cao, Ayman Mohamed, Yih-Kuen Jan

**Affiliations:** 1 Department of Biomedical Engineering, Xi’an Technological University, Xi’an 710021, China; 2 Department of Kinesiology and Community Health, University of Illinois at Urbana-Champaign, Champaign, IL 61820, USA; 3 Department of Sports Science and Physical Education, Tsinghua University, Beijing 100084, China

**Keywords:** local cooling, multiscale entropy, pressure ulcers, regularity, spinal cord injury

## Abstract

Pressure ulcers are one of the most common complications of a spinal cord injury (SCI). Prolonged unrelieved pressure is thought to be the primary causative factor resulting in tissue ischemia and eventually pressure ulcers. Previous studies suggested that local cooling reduces skin ischemia of the compressed soft tissues based on smaller hyperemic responses. However, the effect of local cooling on nonlinear properties of skin blood flow (SBF) during hyperemia is unknown. In this study, 10 wheelchair users with SCI and 10 able-bodied (AB) controls underwent three experimental protocols, each of which included a 10-min period as baseline, a 20-min intervention period, and a 20-min period for recovering SBF. SBF was measured using a laser Doppler flowmetry. During the intervention period, a pressure of 60 mmHg was applied to the sacral skin, while three skin temperature settings were tested, including no temperature change, a decrease by 10 °C, and an increase by 10 °C, respectively. A multiscale entropy (MSE) method was employed to quantify the degree of regularity of blood flow oscillations (BFO) associated with the SBF control mechanisms during baseline and reactive hyperemia. The results showed that under pressure with cooling, skin BFO both in people with SCI and AB controls were more regular at multiple time scales during hyperemia compared to baseline, whereas under pressure with no temperature change and particularly pressure with heating, BFO were more irregular during hyperemia compared to baseline. Moreover, the results of surrogate tests indicated that changes in the degree of regularity of BFO from baseline to hyperemia were only partially attributed to changes in relative amplitudes of endothelial, neurogenic, and myogenic components of BFO. These findings support the use of MSE to assess the efficacy of local cooling on reactive hyperemia and assess the degree of skin ischemia in people with SCI.

## 1. Introduction

Pressure ulcers are a common complications of a spinal cord injury (SCI) that can significantly impact patients’ overall quality of life and even be life-threatening [1]. The annual incidence and prevalence rates of pressure ulcers in the SCI population are reported to be 21% to 30% and 10.2% to 30%, respectively [2]. Although the exact etiology of pressure ulcers remains unclear, unrelieved prolonged pressure is thought to be the primary causative factor that results in decreased blood flow to the compressed soft tissues and eventually tissue necrosis [3]. Additionally, elevated skin temperature has been identified as a causative factor of pressure ulcers [4,5]. Previous animal studies [4,6] showed that local cooling can reduce tissue damage under surface pressure. Based on these promising results, human studies were conducted to demonstrated that local cooling may reduce skin ischemia under pressure [7,8]. Our previous study [7] demonstrated that pressure with cooling induced a smaller reactive hyperemic response compared to pressure with no temperature change and pressure with heating both in people with SCI and able-bodied (AB) controls. Using wavelet analysis, we further showed that the smaller hyperemic response was attributed to reduced endothelial and neurogenic blood flow controls. 

Reactive hyperemia has been traditionally quantified by time-domain parameters such as peak hyperemia, time to peak hyperemia, recovery time, and total hyperemia [7,8,9]. These parameters provide an intuitionistic description of the response but may exhibit large inter- and intra-subject variations [7,8]. Reactive hyperemia has also been investigated using wavelet-based spectral analysis [7]. It has been revealed that skin blood flow (SBF) oscillations contain six characteristic frequencies between 0.005 and 2 Hz, each of which corresponds to a specific blood flow control mechanism [10,11]. The power or mean amplitude of each frequency component is usually used to assess the activity of the corresponding blood flow control mechanism [7,12]. However, wavelet-based spectrum is incapable of characterizing nonlinear properties of blood flow oscillations (BFO), which are structural properties rather than the magnitude of variability [12]. It has been demonstrated that nonlinear properties of BFO could be a critical aspect for assessing SBF regulation and altered nonlinear properties of BFO are associated with microvascular dysfunction [13,14,15,16,17,18]. In our previous study [17], we investigated SBF responses at the first metatarsal head of diabetics and healthy controls induced by locally applied pressure and heating. A multiscale entropy (MSE) method was used to quantify the degree of regularity of BFO. We observed significant changes in the degree of regularity of BFO from baseline to hyperemia in healthy controls but not in diabetics. These results indicated that MSE is capable of revealing altered skin BFO in the diabetic foot. The use of MSE may improve our understanding of how SBF in response to local cooling in people with SCI.

The objective of the present study was to investigate whether MSE could be used to assess the efficacy of local cooling on reactive hyperemia in people with SCI. To our knowledge, this was the first study to investigate nonlinear properties of BFO under different temperatures in people with SCI. As the regulatory mechanisms of reactive hyperemia are thought to be local [7,19,20], the SBF signals were filtered to contain only endothelial, neurogenic, and myogenic oscillations using the ensemble empirical mode decomposition method [21]. We hypothesized that the regularity degree of BFO would undergo different changes during reactive hyperemia under three conditions, i.e., pressure with no temperature change, pressure with local cooling, and pressure with local heating.

## 2. Methods

### 2.1. Participants 

Ten wheelchair users with SCI and 10 AB controls participated in this study. Their demographic data have been reported in our previous publication [7]. Briefly, the mean age (standard deviation, SD) and mean injury duration (SD) of the participants with SCI were 35.8 (11.0) and 9.7 (3.8) years, respectively, while the mean age (SD) of the AB controls was 9.7 (3.8) years. All the participants with SCI met the following criteria: (1) traumatic injury level between C4 and T5; (2) injury level between A (complete motor and sensory injury) and C (incomplete injury) according to American Spinal Injury Association Impairment Scale; and (3) injury duration longer than 6 months. None of the participants suffered from pressure ulcers on the sacrum, cardiopulmonary diseases, or diabetes, or used medications that may affect microcirculation. This study was approved by a university institutional review board (#14307). Each participant gave informed consent prior to any tests.

### 2.2. Data Collection

**Figure 1:**
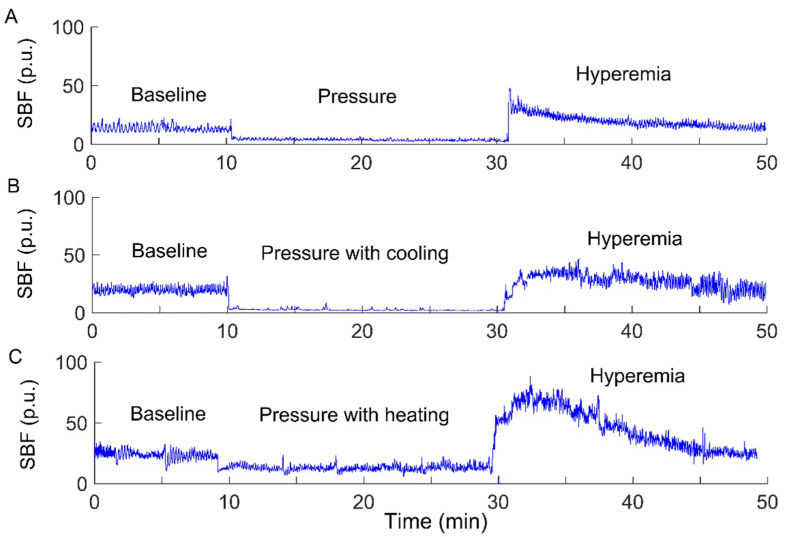
Sacral skin blood flow (SBF) responses to a surface pressure of 60 mmHg without skin temperature changes (**A**), pressure with cooling (**B**), and pressure with heating (**C**) in a participant with a spinal cord injury (SCI). p.u., perfusion unit.

The experiments were carried out in a laboratory where the temperature was kept between 22 and 26 °C. Prior to the tests, the subject stayed in the lab for at least 30 min to achieve a steady level of basal SBF. Then, the subject lay in a prone position on a table. A compound probe (Probe 415–242, Perimed) was attached to the sacral skin, which can be used to heat and cool the skin and simultaneously measure SBF and skin temperature. The subject underwent three experimental protocols, each of which included a 10-min period as baseline, a 20-min intervention period, and a 20-min period for recovering SBF. SBF and skin temperature were recorded by a laser Doppler flowmetry (PeriFlux 5001, Perimed, Ardmore, PA, USA) with a sampling rate of 32 Hz. During the intervention period, a pressure of 60 mmHg was applied to the sacral skin via the probe using a custom-made indenter [7], while skin temperature was maintained constant, decreased by 10 °C, and increased by 10 °C, respectively. The reason for the order of the protocols was that local cooling has less lasting effect on SBF response compared to local heating [7]. To eliminate such lasting effect, two successive protocols were separated by a 30-min washout period [7]. Figure 1 shows typical SBF responses in a subject with SCI under three conditions.

### 2.3. Data Analysis

#### 2.3.1. Modified Sample Entropy

For a time series having N points, {x(i),i=1,…,N}, its sample entropy, denoted as $E_{s}(m,r,N)$, is defined as the negative natural logarithm of the conditional probability that two different sequences within a tolerance r for m points remain within the tolerance for an additional point [22]. $E_{s}$ quantifies the degree of regularity of time series. A smaller $E_{s}$ value indicates a higher degree of regularity and vice versa. In the $E_{s}$ algorithm, a m-point sequence is defined as

(1) $$x_{m}(i)=\{x(i+k),0\leq k\leq m-1\},1\leq i\leq N-m.$$

The distance between two sequences $x_{m}(i)$ and $x_{m}(j)$ is typically defined as

(2) $$d[x_{m}(i),x_{m}(j)]=\max\{|x(i+k)-x(j+k)|,0\leq k\leq m-1,i\neq j\}.$$

If $d[x_{m}(i),x_{m}(j)]\leq r$, the two sequences are considered to be within r. Usually, the parameter r is set to be proportional to the SD of the time series, e.g., 0.15–0.2 × SD [22].

A problem with $E_{s}$ is its dependence on the sampling rate [15]. When the sampling rate is far higher than the frequency of the target dynamics, $E_{s}$ may not quantify the regularity degree of the dynamics and thus yield misleading results. To solve this problem, we proposed a modified algorithm [15], in which a m-point sequence is defined as

(3) $$x_{m}^{\tau }(i)=\{x(i+k\tau ),0\leq k\leq m-1\},1\leq i\leq N-m\tau ,$$

where τ is a lag. Its optimal value could be chosen as the lag corresponding to the first minimum of the auto mutual information function of the time series [15]. The distance between $x_{m}^{\tau }(i)$ and $x_{m}^{\tau }(j)$ is therefore defined as

(4) $$d[x_{m}^{\tau }(i),x_{m}^{\tau }(j)]=\max\{|x(i+k\tau )-x(j+k\tau )|,0\leq k\leq m-1,|j-i|>\tau \},$$

where, the constraint condition |j−i|>τ serves to reduce the influence of the correlation between $x_{m}^{\tau }(i)$ and $x_{m}^{\tau }(j)$ on entropy estimation. Our previous study [15] has demonstrated that the modified sample entropy, denoted as $E_{ms}(m,r,\tau ,N)$, does not depend on the sampling rate both for simulated signals and SBF data. For more details regarding the improvement of $E_{ms}$ over $E_{s}$, see [15,17].

#### 2.3.2. Multiscale Entropy Method

Although $E_{s}$ is frequently referred to as a measure of complexity of time series [22], it is actually a measure of the degree of regularity at a single scale by quantifying the appearance of repetitive patterns [23]. The motivation for assessing the complexity of biological signals is based on the hypothesis that complexity reflects the biological system’s adaptive capacity in an ever-changing environment and that loss of complexity may be an intrinsic feature of pathologic status [23]. It is also hypothesized that biological systems’ operations in response to stimuli involve multiple spatial and temporal scales and thus the complexity of their output variables should be multiscale [23]. In this context, various MSE algorithms were proposed, which generally consist of two procedures, i.e., deriving a group of new series from the original one and computing an entropic measure for each new series. In the original MSE algorithm [24], for a specific scale factor τ, the new series is composed of the mean values of the non-overlapping segments with a length of τ of the original time series. Then, $E_{s}$ is computed for the new series using a constant tolerance r. It has been recognized that this algorithm has several drawbacks [25]. First, the procedure of deriving new series can be seen as a process consisting of low-pass filtering the original time series and down sampling the filtered time series. It was reported that this procedure produces artifacts because the frequency response of the filter exhibits side lobes in the stop band [26]. Second, the parameter r remains constant for all scales, while the SD of the new series may become smaller for larger scales because of the low-pass filtering procedure. This leads to more and more pairs of sequences being within r, thus yielding decreasing entropy values. Additionally, for a scale factor τ, the length of the new series is equal to the length of the original time series divided by τ. Hence, at large scales, the new series may be too short for reliable entropy estimation.

To solve the above drawbacks of the original MSE algorithm, a number of modified MSE methods have been proposed, which have been discussed in [25]. Most of them employed alterative coarse-graining procedures and/or entropic measures. For instance, Zandiyeh and von Tscharner [27] proposed a method called reshape scale to construct new series. It possesses an advantage that the newly constructed time series and the original one has the same length, making it possible to obtain reliable entropy estimations when applying to short time series. Our previous study [17] demonstrated that for SBF signals, $E_{ms}$ is almost identical to $E_{s}$ of the new series constructed by the reshape scale method. Additionally, the results of surrogate tests implied that the combination of the reshape scale method and fuzzy entropy [28] does not have any superiority over $E_{ms}$ for the assessment of the complexity of BFO. In the following, we further demonstrate that $E_{ms}$ of a signal is associated with the relative amplitudes of the frequency components of the signal.

**Figure 2:**
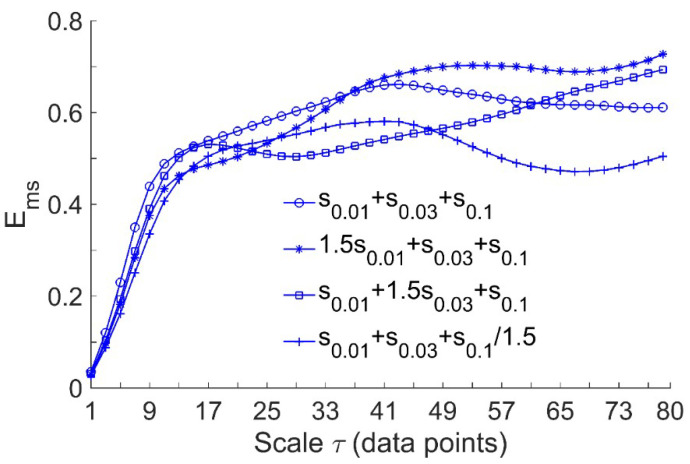
Multiscale entropy $E_{ms}(m,r,\tau ,N)$ of the simulated signals, where $s_{0.01}$, $s_{0.03}$, and $s_{0.1}$ represent sin(2π⋅0.01t), sin(2π⋅0.03t), and sin(2π⋅0.1t) sampled at 32 Hz, respectively. The length of the signals is N = 9600, corresponding to a 5-min period. In the computation of $E_{ms}$, the parameters m = 2 and r = 0.2 × SD were used.

Since the present study aimed to assess the efficacy of local cooling on reactive hyperemia, while the regulatory mechanisms of reactive hyperemia are thought to be local [7,19,20], we performed the following simulation tests. Considering the signals $x_{0}=s_{0.01}+s_{0.03}+s_{0.1}$, $x_{1}=1.5s_{0.01}+s_{0.03}+s_{0.1}$, $x_{2}=s_{0.01}+1.5s_{0.03}+s_{0.1}$, and $x_{3}=s_{0.01}+s_{0.03}+s_{0.1}/1.5$, where $s_{0.01}$, $s_{0.03}$, and $s_{0.1}$ represent sin(2π⋅0.01t), sin(2π⋅0.03t), and sin(2π⋅0.1t) with a sampling interval of 0.313 s (corresponding to the sampling rate of 32 Hz), respectively. As the endothelial, neurogenic, and myogenic oscillations of SBF are around 0.01, 0.03, and 0.1 Hz, respectively, $x_{1}$, $x_{2}$, and $x_{3}$ were used to simulate BFO with augmented endothelial component, augmented neurogenic component, and attenuated myogenic component, respectively. We computed $E_{ms}$ for the signals at scales τ = 1–80. The rationale for this scale range is as follows. As mentioned earlier, when computing $E_{ms}$ at a single scale, the optimal value of τ could be determined by the first minimum of the auto mutual information function of the signal [15]. By this approach, the obtained optimal values of τ for $x_{0}$, $x_{1}$, $x_{2}$, and $x_{3}$ were around 80. On the other hand, we computed the optimal values of τ for the filtered SBF signals that contain only endothelial, neurogenic, and myogenic oscillations. The filtered signal was obtained by decomposing the original SBF signal into a set of mode functions using the ensemble empirical mode decomposition method [21] and then accumulating the mode functions with frequencies between 0.0095 and 0.15 Hz. The filtered SBF signals yielded a wide range of optimal values of τ, which are listed in Table 1. As the overall mean of the τ values was close to 80, it is reasonable to choose 80 as the largest scale for computing $E_{ms}$ of the simulated signals. As shown in Figure 2, $E_{ms}(m,r,\tau ,N)$ exhibits distinct differences among the four signals at large scales. This implies that for SBF signals, changes in relative amplitudes of endothelial, neurogenic, and myogenic oscillations can lead to changes in $E_{ms}$ at large scales.

#### 2.3.3. Multiscale Entropy of SBF Data

In order to investigate the efficacy of local cooling on reactive hyperemia, we computed $E_{ms}$ for the filtered SBF signals during baseline and hyperemia under three conditions. As the duration of reactive hyperemia varied considerably across the protocols (see Figure 1), as well the participants but was longer than 5 min in most cases, $E_{ms}$ was computed for the 5-min segment of the filtered SBF signal starting at the peak hyperemia. The scale range τ = 1–80 and the parameters m = 2 and r = 0.2 × SD were adopted.

### 2.4. Relative Wavelet Amplitude of BFO

In order to understand how the amplitudes of endothelial, neurogenic, and myogenic oscillations influence $E_{ms}$, we computed their relative wavelet amplitudes ($A_{r}$) [29]. Briefly, after performing continuous wavelet transform of the original SBF signal during baseline and the recovery period (31–50 min), the absolute wavelet coefficients were averaged over the same time periods for computing $E_{ms}$ and over the frequency ranges 0.0095–0.02 Hz, 0.02–0.05 Hz, and 0.05–0.15 Hz, respectively. Then, the mean amplitudes of the three components were divided by the mean amplitude over the frequency range 0.0095–2 Hz, yielding their relative amplitudes ($A_{r}$).

### 2.5. Statistical Analysis

The within-group differences in $E_{ms}$ and $A_{r}$ between baseline and reactive hyperemia were examined using Wilcoxon signed-rank tests performed in SPSS 22 (SPSS, Chicago, IL, USA). 

## 3. Results

**Figure 3:**
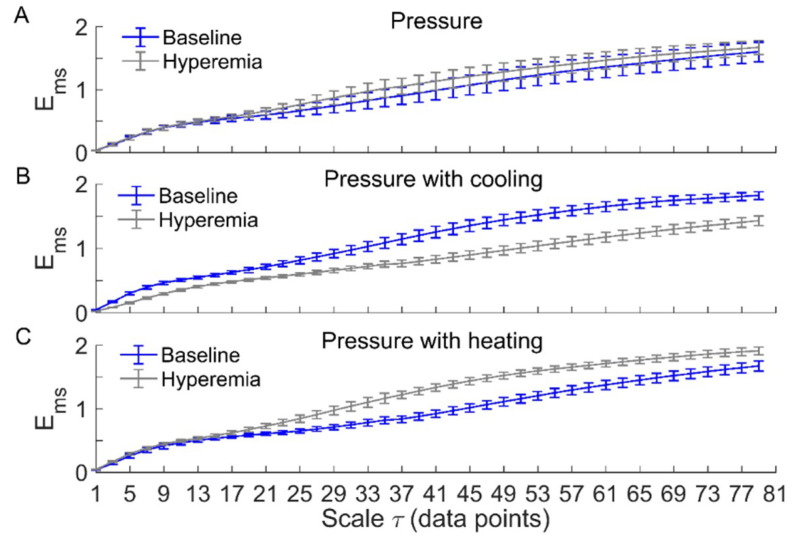
$E_{ms}(m,r,\tau ,N)$ of blood flow oscillations (BFO) associated with endothelial, neurogenic, and myogenic activities in able-bodied (AB) controls during baseline and hyperemia. Data are represented as mean ± standard error. (**A**) Under pressure-induced hyperemia, $E_{ms}$ tended to be higher at large scales compared to baseline, but the differences did not reach a significant level (*p* > 0.05, Wilcoxon signed-rank test). (**B**) During pressure with cooling-induced hyperemia, $E_{ms}$ was significantly lower at all scales (*p* < 0.05) compared to baseline. (**C**) During pressure with heating-induced hyperemia, $E_{ms}$ was significantly higher at the scales τ = 21–73 (*p* < 0.05) compared to baseline.

**Figure 4:**
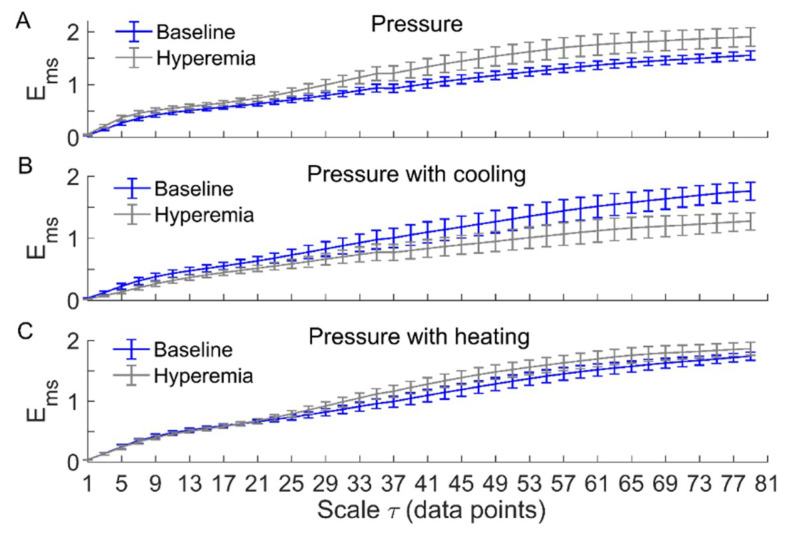
$E_{ms}(m,r,\tau ,N)$ of BFO associated with endothelial, neurogenic, and myogenic activities in people with SCI during baseline and hyperemia. Data are represented as mean ± standard error. (**A**) During pressure-induced hyperemia, $E_{ms}$ was slightly higher at large scales compared to baseline (*p* > 0.05, Wilcoxon signed-rank test). (**B**) During pressure with cooling-induced hyperemia, $E_{ms}$ was lower at the scales τ≥ 23 (*p* = 0.06) compared to baseline. (**C**) During pressure with heating-induced hyperemia, $E_{ms}$ was significantly higher at the scales τ = 31–35 compared to baseline (*p* < 0.05); at the scales τ = 37–65, $E_{ms}$ during hyperemia was also higher compared to baseline (*p* = 0.06).

Figure 3 and Figure 4 compare $E_{ms}$ of BFO between baseline and reactive hyperemia in AB controls and people with SCI, respectively. Under pressure with no temperature change, $E_{ms}$ during hyperemia was slightly higher than that during baseline in both groups, but the differences did not reach the significance level (Figure 3A and Figure 4A). Under pressure with cooling, $E_{ms}$ in AB controls was significantly lower at all scales during hyperemia compared to baseline (*p* < 0.05, Figure 3B), while in people with SCI, $E_{ms}$ was moderately lower during hyperemia at the scales τ≥ 23 compared to baseline (*p* = 0.06, Figure 4B). Under pressure with heating, $E_{ms}$ in AB controls was significantly higher at the scales τ = 21–73 during hyperemia compared to baseline (*p* < 0.05, Figure 3C), while in people with SCI, $E_{ms}$ was significantly higher at the scales τ = 31–35 (*p* < 0.05) and moderately higher at the scales τ = 37–65 (*p* = 0.06) during compared to baseline (Figure 4C).

**Figure 5:**
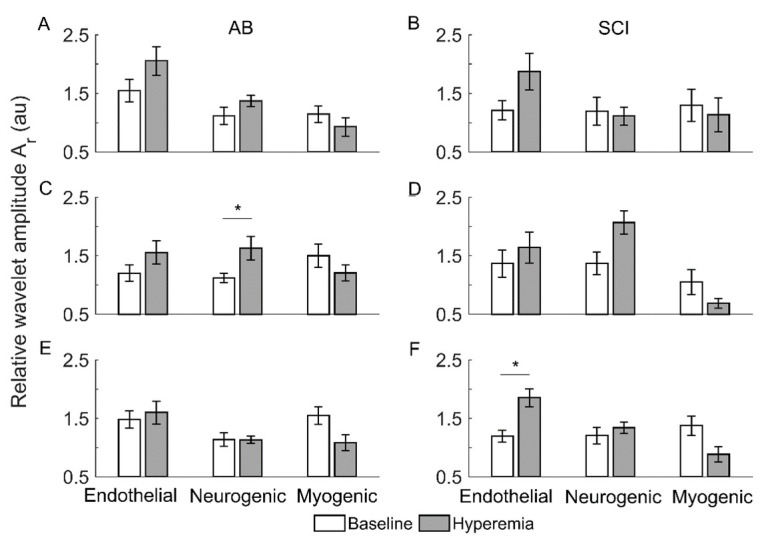
Relative wavelet amplitudes ($A_{r}$) of endothelia, neurogenic, and myogenic oscillations in AB controls and people with SCI during baseline and reactive hyperemia. Data are represented as mean ± standard error. The stars represent *p* < 0.05 (Wilcoxon signed-rank test). (**A**,**B**) The condition of pressure with no temperature change. (**C**,**D**) Pressure with cooling. (**E**,**F**) Pressure with heating.

Figure 5 compares the relative wavelet amplitudes ($A_{r}$) of endothelial, neurogenic, and myogenic oscillations between baseline and reactive hyperemia in two groups. Under pressure with cooling, $A_{r}$ of neurogenic oscillations showed a significant increase from baseline to hyperemia in AB controls (*p* < 0.05, Figure 5C) but not in people with SCI (Figure 5D). Under pressure with heating, $A_{r}$ of endothelial oscillations showed a significant increase from baseline to hyperemia in people with SCI (*p* < 0.05, Figure 5F) but not in AB controls (Figure 5E).

## 4. Discussion

The main findings of the current study are that under pressure with cooling, sacral skin BFO associated with the local mechanisms of SBF regulation were more regular at multiple time scales during reactive hyperemia compared to baseline both in people with SCI and AB controls, whereas under pressure with no temperature change or pressure with heating, BFO were more irregular during hyperemia compared to baseline. These findings support the use of multiscale entropy to assess the efficacy of local cooling on reactive hyperemia and assess the degree of skin ischemia under loading pressure in people with SCI. 

Our results showed that local cooling resulted in a decrease in $E_{ms}$ of BFO from baseline to reactive hyperemia, which was opposite to that resulted from local heating (Figure 3 and Figure 4). Since there is evidence that elevated skin temperature can aggravate pressure-induced skin ischemia [4,5] and local cooling could reduce skin ischemia [7], we deduce that more regular behaviors of BFO during hyperemia are associated with a lower degree of skin ischemia during pressure loading period. This deduction is based on previous studies [7,8]. We observed a smaller hyperemic response under pressure with cooling compared to pressure with no temperature change and particularly pressure with heating in our previous study [7]. As the level of reactive hyperemia is thought to be correlated to the degree of ischemia [30], this observation implied that local cooling could reduce skin ischemia under loading pressure. In the present study, by utilizing multiscale entropy analysis, we further demonstrate that BFO during hyperemia exhibited distinctly different structural features under three conditions. Therefore, the current findings provide a new insight into the association between reactive hyperemia and skin ischemia under surface pressure. 

A recent study by our group [17] demonstrated that $E_{ms}$ is associated with $A_{r}$ of the characteristic frequencies of BFO. Indeed, the simulation results shown in Figure 2 indicate that changes in relative amplitudes of the frequency components of a signal lead to changes in $E_{ms}$ at large scales. Our results indicated that under three conditions, $A_{r}$ of endothelial, neurogenic, and myogenic oscillations exhibited different changes from baseline to hyperemia. Thus, it may be intuitively deduce that changes in $E_{ms}$ of BFO under three conditions were attributed to different changes in $A_{r}$ of endothelial, neurogenic, and myogenic oscillations. However, the differences in $A_{r}$ of the three components between baseline and hyperemia were less distinct as compared to the differences in $E_{ms}$, particularly in AB controls under pressure with cooling and pressure with heating. We therefore speculate that changes in $E_{ms}$ from baseline to hyperemia were also attributed to changes in interactions between the mechanisms of SBF regulation.

**Figure 6:**
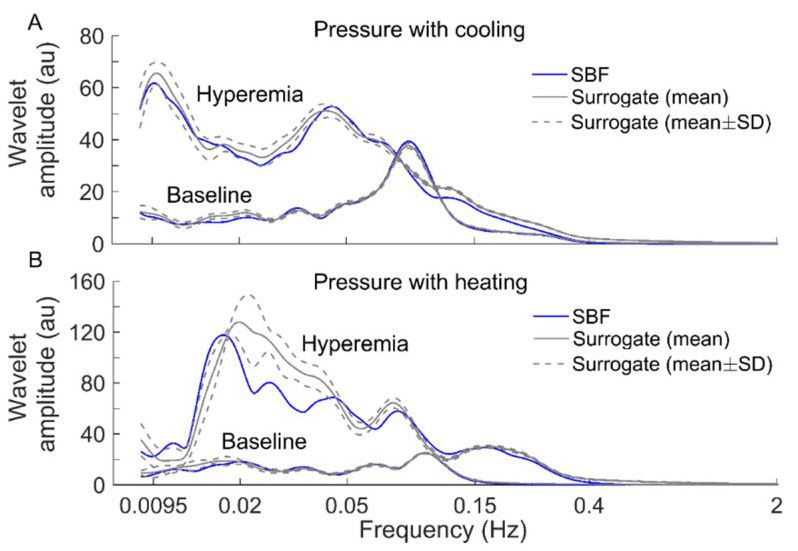
Comparisons of wavelet amplitude spectrum between filtered SBF signals (containing only endothelial, neurogenic, and myogenic oscillations) from an AB control and surrogate time series. For each segment of the SBF signals during baseline and hyperemia, 30 surrogate time series were generated. The spectra of the surrogate time series are presented as mean ± SD. (**A**) Under pressure with cooling. (**B**) Under pressure with heating.

**Figure 7:**
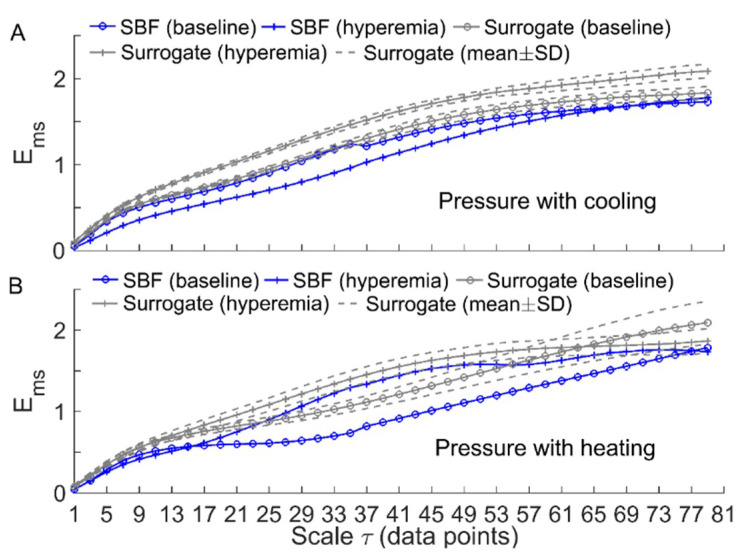
Comparisons of $E_{ms}(m,r,\tau ,N)$ between the filtered SBF signals and surrogate time series (the wavelet amplitude spectra are shown in Figure 6). For each segment of the SBF signals during baseline and hyperemia, $E_{ms}$ was computed for 30 surrogate time series and the results are presented as mean ± SD. (**A**) Under pressure with cooling. (**B**) Under pressure with heating.

To verify the above speculation, we performed the following surrogate tests. Considering two filtered SBF signals (containing endothelial, neurogenic, and myogenic oscillations only) from an AB control under pressure with cooling and pressure with heating, for each segment of the filtered signals during baseline and hyperemia, 30 surrogate time series were generated using the iterated amplitude adjusted Fourier transform method [31]. Theoretically, the surrogate time series was composed of random numbers but preserved the power spectrum of the original signal [31]. As shown in Figure 6 and Figure 7, although the surrogate data and the original signal had similar wavelet amplitude spectra (Figure 6), $E_{ms}$ exhibited distinct differences between them (Figure 7). This suggested that changes in $E_{ms}$ of BFO from baseline to hyperemia were attributed to not only changes in relative amplitudes of endothelial, neurogenic, and myogenic oscillations, but also changes in interactions between the underlying mechanisms. Specifically, under pressure with cooling, $E_{ms}$ of BFO during hyperemia was much lower than those of the surrogate time series (Figure 7A). On the contrary, under pressure with heating, $E_{ms}$ of BFO during hyperemia was similar to those of the surrogate time series (Figure 7B). Hence, we speculate that local cooling and local heating respectively lead to an attenuation and an intensification of the interactions between the mechanisms of SBF regulation during hyperemia. 

Our results also showed that except for the condition of pressure with cooling in AB controls, $E_{ms}$ of BFO showed negligible difference between baseline and hyperemia at small scales (Figure 3 and Figure 4). The reason was that BFO contained only endothelial, neurogenic, and myogenic components with a frequency range of 0.0095–0.15 Hz, while the sampling rate was 32 Hz. As the dominant frequencies of BFO were much lower than the sampling rate, $E_{ms}$ at the small scales did not reflect the structural properties of BFO. In this study, we used a scale range of τ = 1–80 data points based on the observation that for most of our data sets, the optimal value of τ determined by the first minimum of the auto mutual information function of the signal was smaller than but close to 80. As expected, $E_{ms}$ showed only slight changes at the scales close to 80 (Figure 3 and Figure 4), implying that it is unnecessary to compute $E_{ms}$ at larger scales. 

It should be noted that under pressure with cooling and pressure with heating, $E_{ms}$ of BFO in people with SCI underwent smaller changes from baseline to hyperemia compared to AB controls (Figure 3B,C and Figure 4B,C). Additionally, under pressure with cooling, $A_{r}$ of neurogenic oscillations in people with SCI underwent a smaller increase from baseline to hyperemia compared to AB controls (Figure 5C,D). These observations are consist with our previous study [7], in which a weaker reactive hyperemic response was observed in people with SCI. Since it has been found that sensory nerves are involved in reactive hyperemia [32], we speculate that in people with SCI, impaired sensory nerve function contributed to not only diminished hyperemia but also to altered interactions between the mechanisms of SBF regulation during hyperemia. Nevertheless, our results showed that in people with SCI, local cooling also resulted in opposite changes in $E_{ms}$ from baseline to hyperemia compared to local heating, suggesting that local cooling could reduce skin ischemia under loading pressure.

There were several limitations in this study. First, this study had a small sample size, involving only 10 wheelchair users with SCI and 10 AB controls. However, our main purpose was to investigate the feasibility of using multiscale entropy to assess the efficacy of local cooling on reactive hyperemia in people with SCI. Our results showed that multiscale entropy could reveal differences in the structural properties of BFO during reactive hyperemia under three conditions, which cannot be revealed by time- or frequency-domain parameters. Future study may need to recruit more participants to validate our findings. Second, because for several subjects, there were variations in $E_{ms}$ and $A_{r}$ of BFO during baseline across three protocols, we were unable to examine the within-subject differences in $E_{ms}$ and $A_{r}$ between three conditions and the between-subject differences. The reasons for the variations of basal BFO across the protocols might be that the lasting effect of the stimulus, i.e., pressure or pressure with cooling, was not completely eliminated during the washout period and/or the subjects’ posture underwent changes due to fatigue. Nevertheless, our main purpose has been achieved by examining the within-subject differences in $E_{ms}$ between hyperemia and baseline under three conditions.

## 5. Conclusions

Our results showed that under pressure with cooling, sacral skin BFO associated with the local mechanisms of SBF regulation both in people with SCI and AB controls were more regular at multiple time scales during reactive hyperemia compared to baseline, whereas under pressure with no temperature change particularly pressure with heating, BFO were more irregular during hyperemia compared to baseline. Additionally, the results of surrogate tests indicated that changes in degree of regularity of BFO from baseline to hyperemia were only partially attributed to changes in relative amplitudes of endothelial, neurogenic, and myogenic oscillations. These findings support the use of multiscale entropy to assess the efficacy of local cooling on reactive hyperemia and assess the degree of skin ischemia under loading pressure.

## Figures and Tables

**Table 1:** Optimal values of τ for the filtered SBF signals.

	Pressure with No Temperature Change		Pressure with Cooling		Pressure with Heating	
	Baseline	Hyperemia	Baseline	Hyperemia	Baseline	Hyperemia
AB	84.4 ± 8.2	77.4 ± 18.7	77.5 ± 12.2	84.3 ± 9.5	80.5 ± 15.4	64.7 ± 10.3
SCI	78.9 ± 12.0	62.4 ± 17.3	67.3 ± 14.3	56.7 ± 25.5	79.8 ± 12.4	67.2 ± 12.0

Data are represented as mean ± standard deviation (SD). The overall mean and SD are 73.6 and 15.4, respectively.
